# Spinal cord gray matter atrophy is associated with disability in spinal muscular atrophy

**DOI:** 10.1007/s00415-024-12740-3

**Published:** 2025-01-07

**Authors:** Eva Maria Kesenheimer, Maria Janina Wendebourg, Claudia Weidensteiner, Laura Sander, Matthias Weigel, Tanja Haas, Dirk Fischer, Christoph Neuwirth, Nathalie Braun, Markus Weber, Cristina Granziera, Michael Sinnreich, Oliver Bieri, Regina Schlaeger

**Affiliations:** 1 Department of Neurology, University Hospital Basel, University of Basel, Basel, Switzerland; 2 Department of Clinical Research, University Hospital Basel, University of Basel, Basel, Switzerland; 3 Translational Imaging in Neurology (ThINk), Department of Biomedical Engineering, University of Basel, Basel, Switzerland; 4 Clinic for Neurorehabilitation and Paraplegiology, REHAB Basel, Basel, Switzerland; 5 Division of Radiological Physics, Department of Radiology, University Hospital Basel, Basel, Switzerland; 6 Research Center for Clinical Neuroimmunology and Neuroscience Basel (RC2NB), University of Basel, Basel, Switzerland; 7 Division of Neuropediatrics and Developmental Medicine, University Childrens` Hospital of Basel (UKBB), University of Basel, Basel, Switzerland; 8 Neuromuscular Diseases Unit/ALS Clinic, Kantonsspital St. Gallen, St. Gallen, Switzerland; 9 Department of Biomedical Engineering, University of Basel, Allschwil, Switzerland; 10 Department of Biomedicine, University of Basel, Basel, Switzerland

**Keywords:** Spinal muscular atrophy, SMA, Magnetic resonance imaging, Spinal cord atrophy, Spinal cord imaging

## Abstract

**Background:**

With the approval of disease-modifying treatments for 5q-spinal muscular atrophy (SMA), there is an increasing need for biomarkers for disease course and therapeutic response monitoring. Radially sampled Averaged Magnetization Inversion Recovery Acquisitions (rAMIRA) MR-imaging enables spinal cord (SC) gray matter (GM) delineation and quantification in vivo. This study aims to assess SC GM atrophy in patients with 5q-SMA and its associations with clinical disability.

**Methods:**

Twenty-one patients with 5q-SMA and twenty-one age- and sex-matched healthy controls (HCs) prospectively underwent 3 T axial 2D-rAMIRA MR-imaging at the intervertebral disc levels C2/C3-C5/C6 and T_max_ (lumbar enlargement level). Associations between SC GM areas with muscle strength tested by dynamometry, Motor Function Measure (MFM), revised upper limb module (RULM), Revised Hammersmith Scale (RHS), and SMA-Functional Rating Scale (SMA-FRS) were assessed by Spearman Rank correlations and linear regression analysis.

**Results:**

Compared to HCs, patients had significantly reduced SC GM areas at levels C3/C4 (relative reduction (RR) = 13.6%, p < 0.0001); C4/C5 (RR = 16.7%, p < 0.0001), C5/C6 (RR = 17.1%, p < 0.0001), and T_max_ (RR = 17.4%, p < 0.0001). Significant correlations were found between cervical SC GM areas and muscle strength, RULM, MFM, RHS, and SMA-FRS. In linear regression analysis, GM area C3/C4 explained 33% of RHS variance.

**Conclusion:**

SC GM atrophy is detectable in patients with 5q-SMA and is consistently associated with clinical measures of upper limb function, physiotherapeutic assessments, and SMA-FRS indicating the clinical relevance of the observed atrophy. Further longitudinal investigations are necessary next steps to evaluate this novel and easily applicable imaging marker as a potential disease course and therapeutic response marker.

**Supplementary Information:**

The online version contains supplementary material available at 10.1007/s00415-024-12740-3.

## Introduction

Spinal muscular atrophy (SMA) is a genetically determined neurodegenerative disorder characterized by progressive loss of motor neurons in the spinal cord (SC) and brain stem secondary to mutations in the survival motor neuron gene [1–3]. SMA is one of the most devastating neurological diseases leading to progressive muscle weakness, mobility impairment and often severe respiratory, gastrointestinal and orthopedic complications, and often reduced life span [4–6]. Substantial advances in understanding SMA etiopathogenesis have catalyzed the development of novel therapeutic strategies [7–12]. With the approval of the first disease-modifying treatments for SMA, the need for biomarkers that allow reliable monitoring of the disease course and the therapeutic response in patients with SMA has risen [13].

Until recently, the SC gray matter (GM) and white matter (WM) could not be reliably evaluated with Magnetic Resonance Imaging (MRI) due to technical challenges such as the small diameter of the SC and artifacts from physiological motion as well as from the surrounding tissues. Novel developments of MRI-based techniques now enable a quantitation of SC GM and WM in vivo [14–18]. SC GM atrophy is known to have a clinical impact in multiple sclerosis [19–21] and can already be detected in the earliest stages [22]. Latest advances in MRI sequence development (rAMIRA, radially Averaged Magnetization Inversion Recovery Acquisitions method) allow an enhancement and fine-tuning of the SC tissue contrast [23, 24]. In Post-Polio-Syndrome, a putative “pure” lower motor neuron disorder, significant atrophy of the cervical and thoracic SC GM was found based on rAMIRA imaging with significant segment-wise associations between GM area and muscle force in corresponding myotomes [25].

Currently, it is unclear whether SC GM atrophy can be reliably detected in patients with SMA and could serve as a potential maker of the disease course and therapeutic outcome. The few existing publications using a T2* contrast show equivocal results [26, 27].

The first aim of this study was to investigate cervical and thoracic SC GM areas using rAMIRA imaging in patients with genetically confirmed 5q-SMA II and III in cross-sectional comparison to sex- and age-matched healthy controls (HCs). The second objective was to evaluate level-wise associations of GM atrophy with muscle force in the corresponding myotome assessed by handheld dynamometer and associations with established measures of patient reported disability (SMA-FRS) [28] and motor performance in SMA (Revised Upper Limb Module (RULM) [29], Revised Hammersmith Scale (RHS) [30, 31], and Motor Function Measure (MFM) [32, 33]).

## Methods

### Recruitment and inclusion

This study was a single-center prospective observational case–control study conducted at the University Hospital Basel in Switzerland. Patients were investigated at the Neuromuscular Competence Center, University Hospital Basel between 08/2020 and 04/2022.

Inclusion criteria were age > 11 years and genetically confirmed 5q-SMA Type II or III for patients. Exclusion criteria included active neuromuscular or other neurological conditions explaining the symptoms or interfering with the examinations, other severe chronic disease, pregnancy, and contraindications for MRI scanning. Thirty-seven patients with 5q-SMA were screened, and twenty-one patients were included in this study (Supplemental material Fig. 1). Twenty-one healthy controls were also included, who were selected to be age- and sex-matched on a case-by-case basis.

**Fig. 1:**
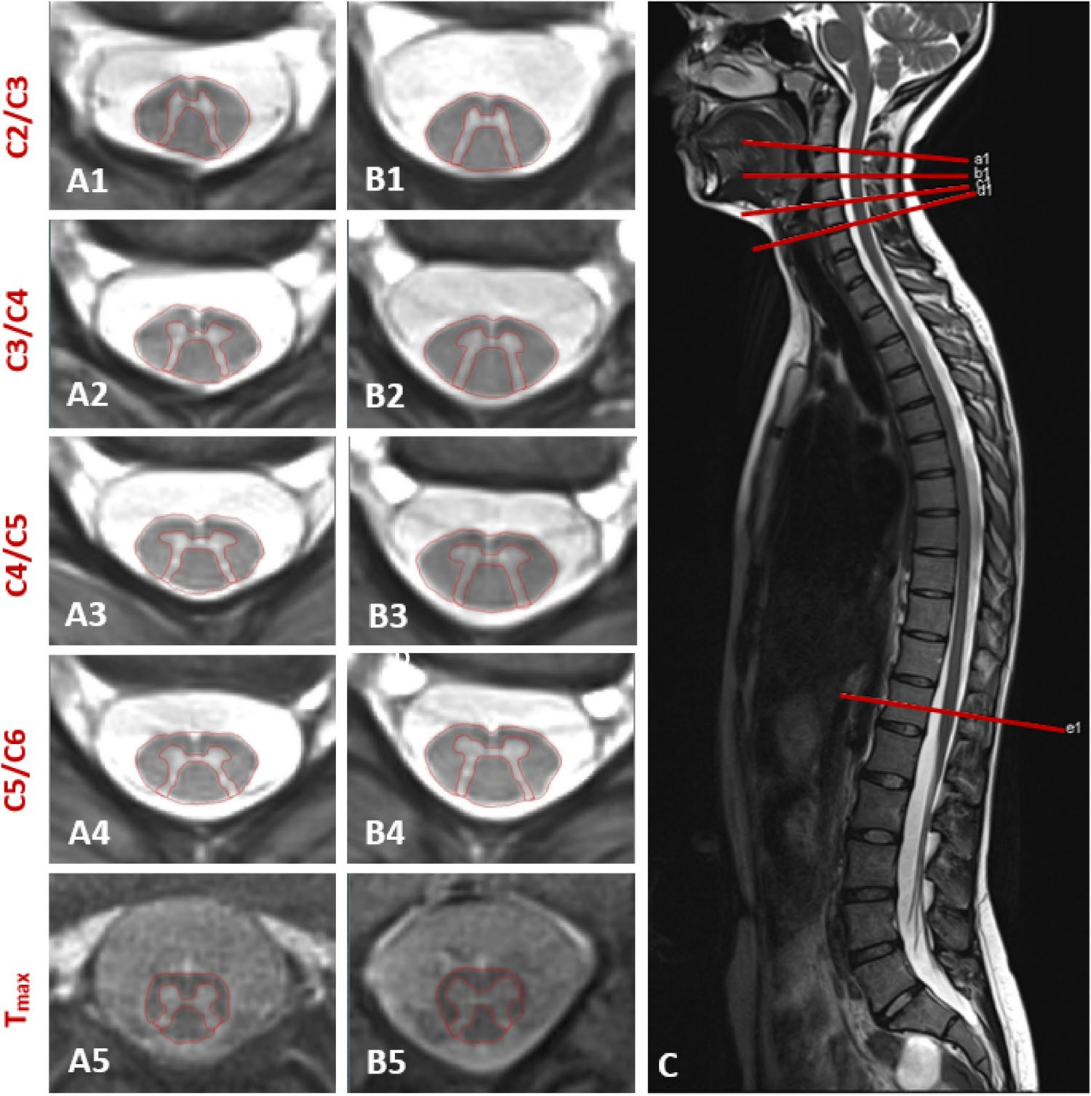
Axial 2D rAMIRA imaging at the intervertebral disc levels C2/C3-C5C/6 and T_max_ (level of the lumbar enlargement) (axial in-plane resolution 0.5 × 0.5mm2, slice thickness 8 mm) of an SMA patient (19 years, female) (A1–A5) compared to her age- and sex-matched HC (B1–B5). C shows the T2 sagittal overview of the spinal cord with the planning of the axial images, which are acquired perpendicular to the cervical spinal cord

Since we had no clear expectation about the differences in rAMIRA-derived GM area between patients with SMA II and III and HCs, a formal sample size calculation was not possible. In a similar study based on the same rAMIRA imaging method in 20 patients with a different lower motor neuron disorder, the post-polio syndrome, and 20 HCs, a significant difference in baseline SC GM area between patients and HCs could be shown, e.g., of 3.05mm^2^ (relative reduction of 17.1%) at the intervertebral disc level C3/C4, and a significant difference at T_max_ of 3.96 mm^2^ (relative reduction of 19.3%) [25].

## MR image acquisition

All participants were examined on the same 3 T MR system (Magnetom PRISMA, Siemens Healthineers, Erlangen, Germany) at the University Hospital Basel using a 64-channel head and neck coil and a built-in spine coil. We performed axial 2D rAMIRA imaging perpendicular to the SC at selected levels at the intervertebral disc levels C2/C3-C5/6 and T_max_ (level of the lumbar enlargement-visually determined at the widest sagittal diameter of the lower thoracic SC) (Fig. 1, Image C). A1–A5 show the axial rAMIRA images at the intervertebral disc levels C2/C3–C5/C6 and T_max_ of a patient with SMA compared to her age- and sex-matched HC (B1-B5).

We chose these levels, based on considerations of technical feasibility, reproducibility (disc level), and SMA phenotype (predominant involvement of proximal arm and leg muscles that are innervated by motor neurons situated close to the cervical/lumbar enlargements in the spinal cord). Anatomically axial SC GM size varies across the different disc levels, with largest gray matter areas found at the levels of the cervical and lumbar spinal cord enlargements. These project approximately to the disc levels C3/C4-C4/C5 and T_max_.

For axial SC imaging and quantification, we used a rAMIRA-based imaging protocol [23]. The rAMIRA sequence consists of an initial inversion recovery preparation followed by a cine-like balanced steady-state free precession (bSSFP) readout module. Several inversion images with different tissue contrast are acquired. In this study, the acquisition parameters were: mean inversion times TI_eff_ = 174ms, 239ms, 304ms, 368ms, 433ms, field of view (FOV) = 128 × 128mm^2^, 512 readout samples (included 2 × oversampling), 260 radial projections, 10 segments, isotropic in-plane resolution 0.50 × 0.50mm^2^, slice thickness 8mm, TR_bSSFP_ = 6.49ms (“echo spacing”), TE_bSSFP_ = 3.23ms, excitation flip angle 50°, receiver bandwidth = 310 Hz/px, and averages = 2. Cardiac triggering was performed on every 3rd to 4th heartbeat using a standard infrared finger clip to mitigate potential pulsation artifacts. The overall TR of the rAMIRA sequence depended on the pulse rate and was kept around 3s. For a heartbeat of 60 beats per minute (bpm), the resulting acquisition time (TA) corresponded to TA = 2:39min per slice. One transversal slice of thickness 8 mm orientated orthogonal to the course of the SC was acquired at each level. In total, four slices were acquired in the cervical SC, and one slice in the thoracic SC, leading to a total TA of around 13-14min. The five inversion images at each level were averaged to yield a morphological SC image with high GM-to-WM-contrast.

## MRI analysis

TH and EMK systematically checked the quality of the rAMIRA images for the presence of artifacts: The TC area at T_max_ (lumbar enlargement level) in two HCs could not be segmented due to movement artifacts. In five patients with SMA, thoracic imaging was not possible due to osteosynthesis material. In one of these patients, the cervical levels C4/C5 and C5/C6 and in one further patient the intervertebral disc level C4/C5 were imaged but could not be segmented due to artifacts by osteosynthesis material.

Segmentation of the SC cross-sectional areas was performed semi-automatically using the software JIM 7 (www.xinapse.com). SC GM was segmented manually. WM area was calculated as the subtraction of GM area from TC area. This approach was described in detail before [20, 25].

The inter-rater reliability of the manual segmentations, determined by two independent raters (EMK and MJW), was excellent. The raters were masked regarding disease vs. HC status. Intra-class correlation coefficients (ICCs, two-way random, absolute agreement) were 0.92–0.97 for TC areas (single measures intraclass correlation) and 0.85–0.94 for GM areas (average measures intraclass correlation) [34].

To reduce anatomical variability, normalization of SC GM areas was performed following the procedure described in Kesenheimer et al. and Papinutto et al. [35–37] based a) on the product of the anterior–posterior and lateral spinal canal diameter at the intervertebral disc levels C3/C4 and C4/C5 alone and on these products each with b) sex and total intracranial volume, and c) together with white matter volume. As these normalizations did not substantially reduce variability in patients, we present non-normalized data in the following result section.

## Clinical and laboratory assessments

All participants were clinically/neurologically investigated at the Neuromuscular Competence Center, University Hospital Basel. Quantitative muscle force tests by hand dynamometer (MicroFet 2, Hoggan Scientific, Salt Lake City, Utah, USA) of selected muscles following the ENCALS protocol were performed, and muscle strength was reported in Newton [38]. All patients completed the SMA functional rating scale [28] and physiotherapeutic assessments with Motor Function Measure (MFM) [32, 33], Revised Hammersmith Scale (RHS) for SMA [30, 31], and Revised Upper Limb Module (RULM) [29].

The MFM is a validated assessment tool for patients with neuromuscular diseases, evaluating motor function across three functional dimensions: standing position and transfers, axial and proximal motor function, and distal motor function.

The RULM is specifically designed to measure upper limb function and excels in identifying changes in motor function in non-ambulatory patients in SMA. The RHS primarily assesses gross motor functions of the axial and lower limb muscles.

These tests have been used as outcome measures in pivotal clinical trials in SMA [11, 12]. These assessments were performed by experienced and trained physiotherapists.

## Statistical analysis

We used SPSS version 28 (IBM Corp.) and JMP Pro Version 16 (SAS Institute Inc.) for data analysis.

The aim of the primary analysis was to compare rAMIRA-derived SC TC and GM areas between patients with SMA and their age- and sex-matched HCs. Distribution was tested with Kolmogorov–Smirnov and Shapiro–Wilk tests. Acknowledging the small sample size, we used Wilcoxon signed-rank tests for pairwise comparison of SC areas between patients and age- and sex-matched HCs. A p value < 0.05 was considered significant. We also present raw data and means of the SC metrics by SMA subtype.

Secondarily, the association of SC GM area with parameters of motor performance in patients with SMA was assessed in the SC levels with significant SC GM area differences between patients and HCs. Given the relatively small sample size, and the non-normal distribution of some of the muscle strength tests (elbow extension, first dorsal interosseus, and hand grip strength), physiotherapeutic assessments (RULM), and patient reported outcome (SMA-FRS), correlations between SC GM areas and the parameters of motor performance were first investigated by performing Spearman Rank correlation analyses. To adjust for multiple testing in the secondary analysis, we performed a Bonferroni correction.

Linear regression analyses were then performed to assess the associations between SC GM area at the level C3/C4 as predictor variable and those motor outcomes that were normally distributed: MFM and Revised Hammersmith Scale. The intervertebral disc level C3/C4 was chosen for this analysis as this level—based on the results of a cadaveric study [39] and an in vivo MRI study of healthy volunteers [15]—corresponds to the cord segment C4/C5, a cord segment that innervates proximal arm muscles that are predominantly affected in SMA.

## Results

### Demographics and clinical data

Twenty-one patients with SMA and twenty-one age- and sex-matched HCs were included in this study between 08/2020 and 07/2022 after written informed consent was given. Demographics and clinical characteristics of patients with SMA and HCs are shown in Table 1. Patients and HCs were well matched regarding age (mean age / SD in patients 41.3 years / 11.6 vs. HCs 41.7 years / 11.4) and sex (twelve men in both groups). Mean age at symptom onset was 6.4 years, with 14% of patients having a symptom onset before 18 months (SMA type II); 38% before (SMA type IIIa) and 48% after the age of 3 years (SMA type IIIb) [40].

**Table 1:** Clinical characteristics of patients with SMA and healthy controls and results of the physiotherapeutic assessments and SMA-FRS of patients with SMA

	Patients with SMA (*n* = 21)	HCs (*n* = 21)
Sex (male/female)	12:9	12:9
Mean age (SD) (years)	41.3 (11.6)	41.7 (11.4)
Median (Min–max)	44 (18.3–59.3)	44.7 (19.7—58.9)
Mean age at disease onset (SD) (years)	6.2 (5.8)	NA
Median (Min–max)	2.5 (1–17)	
Mean disease duration (SD) (years)	34.8 (12.2)	NA
Median (Min–max)	35.3 (11.3–57.6)	
BMI (SD) (kg/m^2^)	21.6 (3.7)	25.1 (4.6)
Median (Min–max)	21 (15.9–28.4)	24.4 (17.6—33.5)
SMA Type		
Type II	3 (14.3%)	NA
Type IIIa	8 (38.1%)	
Type IIIb	10 (47.9%)	
Achieved milestones at any time		
Sit	1 (4.8%)	NA
Stand	1 (4.8%)	
Walk	19 (90.5%)	
SMA specific treatment		
Nusinersen	12*	NA
Risdiplam	5	
None	5	
Mean revised Hammersmith scale (SD)	30.9 (20.3)	NA
Min–max	0–69	
Mean revised upper limb module (RULM) (SD)	28.1 (10.3)	NA
Min–max	0–37	
Mean motor function measure (MFM) (SD)	64.2 (25.0)	NA
Min–max	7.3–100	
Mean SMA-FRS (SD)	32.2 (14.6)	NA
Median (Min–max)	39 (4–48)	

*One patient switched from Nusinersen to Risdiplam

### SC GM area in patients and HC

Table 2 shows the mean, median, SD, and range of GM areas for patients with SMA and HCs.

**Table 2:** Mean and median of spinal cord gray matter (GM) area, standard deviation (SD), and range of GM areas for patients with SMA and healthy controls

GM area at intervertebral disc level	Group	*n*	Mean GM area (mm^2^)	Median GM area (mm^2^)	SD	Minimum	Maximum
C2/C3	SMA HC	21 21	15.9 16.8	15.7 17.0	1.6 1.6	11.9 14.1	18.9 19.2
C3/C4	SMA HC	21 21	17.1 19.8	17.9 19.5	2.2 1.8	13.0 17.2	20.5 24.2
C4/C5	SMA HC	19 21	18.0 21.6	18.8 22.0	2.2 2.2	14.8 16.6	21.2 25.5
C5/C6	SMA HC	20 21	17.5 21.1	17.4 21.2	2.4 1.5	12.4 16.7	21.8 24.7
T_max_	SMA HC	16 21	22.2 26.4	22.5 25.7	2.5 2.9	16.8 21.3	26.8 33.4

In pairwise comparisons using Wilcoxon signed-rank tests, we found significantly reduced GM areas in patients with SMA compared to HCs at the intervertebral disc levels close to the cervical and lumbar enlargements, namely C3/C4 relative reduction (RR): 13.6%, p < 0.0001; C4/C5 RR: 16.7%, p < 0.0001; C5/C6 RR: 17.1%, p < 0.0001; T_max_ RR 17.4%, p < 0.0001. No significant area reduction was found at the intervertebral disc level C2/C3 (RR 5.4%, *p* = 0.085) (Table 3).

**Table 3:** Mean differences in spinal cord gray matter (GM) areas (in mm2) and relative reductions of GM area (in %) between patients with SMA and their age- and sex-matched healthy controls

GM area at intervertebral disc level	Difference (mm2) in GM areas between patients with and HCs			Relative reductions of GM area (in %)	*p*	*p* (2nd rater)
	(Mean)	(SE)	(95% CI)			
C2/C3	−0.9	0.49	(−2.0, 0.1)	5.4	0.085	0.322
C3/C4	−2.7	0.66	(−4.0, −1.3)	13.6	** < 0.001**	**0.019**
C4/C5	−3.6	0.74	(−5.2, −2.1)	16.7	** < 0.001**	**0.001**
C5/C6	−3.6	0.55	(−4.7, −2.4)	17.1	** < 0.001**	** < 0.001**
T_max_	−4.6	0.95	(−6.6, −2.6)	17.4	** < 0.001**	**0.002**

First and second *p* values were derived from Wilcoxon nonparametric comparisons

Standard error (SE), 95% confidence interval (CI), and gray matter (GM)

Group differences for TC area were still significant at the intervertebral disc levels C2/C3-C5/C6 and T_max_, but less pronounced (see Table 4), but not significant for WM area, in line with the pathophysiology of a lower motor neuron disease.

**Table 4:** Mean differences in total cord (TC) areas (in mm2) and relative reductions of TC area (in %) between patients with SMA and their age- and sex-matched healthy controls

TC area at intervertebral disc level	Difference (mm^2^) in TC areas between patients with SMA and HCs			Relative reductions of TC area (in %)	*p*	*p* (2nd rater)
	(Mean)	(SE)	(95% CI)			
C2/C3	−3.3	2.33	(−8.2, 1.5)	4.0	0.181	0.217
C3/C4	−6.2	2.22	(−10.8, −1.5)	7.1	**0.012**	0.050
C4/C5	−7.2	2.52	(−12.5, −2.0)	8.0	**0.019**	**0.033**
C5/C6	−6.8	1.76	(−10.5, −3.1)	7.9	**0.003**	**0.0100**
T_max_	−5.8	2.00	(−10.1, −1.5)	9.4	**0.016**	**0.013**

First and second *p* values were derived from Wilcoxon nonparametric comparisons

Standard error (SE), 95% confidence interval (CI), and total cord (TC)

**Fig. 2:**
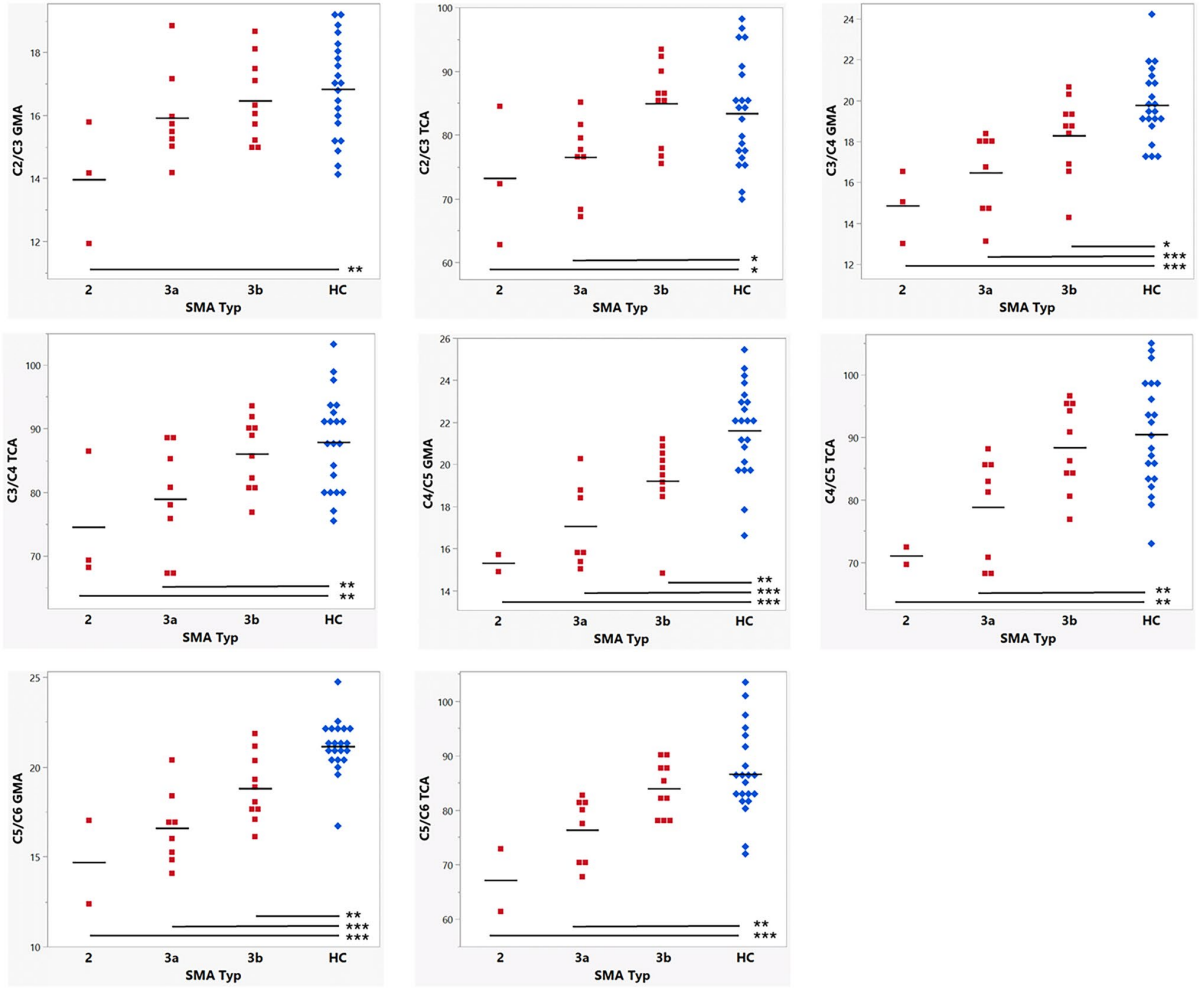
Spinal cord gray matter area (GMA) (in mm^2^) and total cord area (TCA) (in mm^2^) in patients with SMA Type 2, 3a, 3b and healthy controls at the intervertebral disc levels C2/C3-C5/C6 and the level of the lumbar enlargement (T_max_). Mean values are indicated as (─). Group differences (student’s *t*, all pairwise comparisons) are summarized as follows: p values < 0.05 are marked with *, *p* values < 0.01 with **, and *p* values < 0.001 with ***. Note that patients with SMA type 2 could not be imaged at T_max_ due to osteosynthesis material

Patients across all SMA subtypes showed significant SC GM atrophy compared to HCs, with more pronounced SC GM atrophy in the more severe phenotypes (Fig. 2). Due to the small sample size with *n* = 3 for patients with SMA type II, raw data and the mean values are shown (Fig. 2). GM areas showed significantly smaller GM areas in SMA Type IIIa than IIIb at the intervertebral disc level C3/C4-C5/6 and between SMA Type IIIb and HCs at the intervertebral disc levels C3/C4-C5/C6 and T_max_, and the corresponding data for TC area are presented.

### Associations between SC GM area and clinical outcomes

Spearman rank correlations between SC GM area with muscle strength are presented in detail in Table 5: In brief, positive correlations were observed between SC GM areas at all intervertebral disc levels and muscle strength, with Spearman rank correlation coefficients in the range of 0.47—0.73, with one exception: GM area at the most cranial intervertebral disc level C3/C4 and muscle strength of the most distal muscle, the FDI (rho = 0.39, *p* = 0.089).

**Table 5:** Associations between spinal cord gray matter area at corresponding intervertebral disc levels and muscle strength, measured by handheld dynamometry using Spearman Rank Rho

GM area level	Shoulder abduction	Wrist extension	Elbow extension	Hand grip strength	First dorsal interosseous
C3/C4	Rho = 0.52 *p* = 0.017 (*n* = 21)	**Rho = 0.67** ***p* = 0.001** (*n* = 21)	**Rho = 0.61** ***p* = 0.005** (*n* = 19)	**Rho = 0.56** ***p* = 0.009** (*n* = 21)	Rho = 0.39 *p* = 0.089 (*n* = 20)
C4/C5	Rho = 0.48 *p* = 0.036 (*n* = 19)	**Rho = 0.67** ***p* = 0.002** (*n* = 19)	Rho = 0.52 *p* = 0.035 (*n* = 17)	Rho = 0.47 *p* = 0.043 (*n* = 19)	Rho = 0.55 *p* = 0.018 (*n* = 18)
C5/C6	**Rho = 0.59** ***p* = 0.006** (*n* = 20)	**Rho = 0.73** **p < 0.001** (*n* = 20)	**Rho = 0.62** ***p* = 0.007** (*n* = 18)	**Rho = 0.62** ***p* = 0.003** (*n* = 20)	Rho = 0.56 *p* = 0.012 (*n* = 19)

*Rho* Spearman Rank rho

To adjust for multiple muscle strength testing, we performed a Bonferroni correction with a correction factor of five, resulting in a significance level of *p* < 0.01, two-sided *p*

Correlations with *p* < 0.01 are bold

*ns* non-significant with p > 0.20

Spearman rank correlations of SC GM areas with physiotherapeutic assessments are presented in Table 6: cervical SC GM areas at the intervertebral disc levels C3/C4, C4/C5, and C5/C6 were each significantly and positively correlated with upper limb function as measured by RULM.

**Table 6:** Associations between spinal cord gray matter area at a given level and physiotherapeutic assessments as well as SMA-FRS using Spearman Rank Rho

GM area level	MFM total	RULM	Revised Hammersmith Scale	Total SMA-FRS
C3/C4	**Rho = 0.62** ***p* = 0.003** (*n* = 20)	**Rho = 0.65** ***p* = 0.002** (*n* = 21)	**Rho = 0.65** ***p* = 0.001** (*n* = 21)	**Rho = 0.62** ***p* = 0.003** (*n* = 21)
C4/C5	Rho = 0.45 *p* = 0.064 (*n* = 18)	**Rho = 0.58** ***p* = 0.009** (*n* = 19)	Rho = 0.44 p = 0.062 (*n* = 19)	Rho = 0.45 *p* = 0.054 (*n* = 19)
C5/C6	Rho = 0.51 *p* = 0.025 (*n* = 19)	**Rho = 0.62** ***p* = 0.004** (*n* = 20)	Rho = 0.50 *p* = 0.026 (*n* = 20)	Rho = 0.53 *p* = 0.016 (*n* = 20)
T_max_	ns	ns	ns	ns

*Rho* Spearman Rank rho

To adjust for multiple testing, we performed a Bonferroni correction with a correction factor of four, resulting in a significance level of *p* < 0.0125, two-sided *p*. Correlations with *p* < 0.0125 are bold

*ns* non-significant with *p* > 0.20

SC GM area at the intervertebral disc level C3/C4 showed significant positive correlations with MFM, RHS, and SMA-FRS.

SC GMA area at the intervertebral disc levels C4/C5 and C5/C6 showed positive (rho in the range of 0.44—0.53) but borderline non-significant correlations with MFM; RHS and SMA-FRS. We did not find significant correlations between SC GM area at T_max_ and clinical metrics in this cohort.

In linear regression analyses, SC GM area at the intervertebral disc level C3/C4 explained 32% of MFM variance and 33% of RHS variance, respectively (Table 7). In sensitivity analyses, the variables weight and height were subsequently included into the models, but did not improve prediction of a) MFM or b) RHS in this cohort.

**Table 7:** Linear regression analysis with gray matter area at the intervertebral disc level C3/C4 in patients with SMA as predictor variable and physiotherapeutic assessments as outcome

Model	Outcome	Factor	Estimate	SE	*t* value	*p* value
Model 1 Adj. *r*^2^ = 0.31 *p* value = 0.006	MFM total	Intercept GM area C3/C4	−53.79 6.85	38.32 2.21	−1.40 3.10	0.177 **0.006**
Model 2 Adj. *r*^2^ = 0.33 *p* value = 0.004	Revised Hammersmith Scale	Intercept GM area C3/C4	−64.97 5.60	29.62 1.72	−2.19 3.26	**0.041** **0.004**

## Discussion

There is still an unmet need for objective surrogate markers of disease progression in adolescent and adult patients with SMA, in whom motor disability usually worsens very slowly. Challenges in the use of established physiotherapeutic disease markers include susceptibility to daily fluctuations, dependence on patients’ collaboration, and a ceiling-effect (e.g., full score in the RULM before treatment impeding the detection of improvement [41].

The imaging method rAMIRA allows time efficient and easy to perform quantification of SC GM area in vivo with the classical advantages of an MRI surrogate, i.e., objectiveness and robustness regarding fluctuations in motor performance and collaboration. In this study, patients with SMA types II and III showed significant SC GM, but not WM atrophy in both the cervical and lower thoracic SC compared to age- and sex-matched HCs. Atrophy was particularly pronounced at the level C3/C4-C5/6 and at the lumbar enlargement T_max_, reflecting the phenotype of the disease with predominant weakness of proximal muscles.

The non-significant spinal cord gray matter reduction of −5.4% observed at the upper most level C2/C3 (projecting to the cord segment C3/C4 that contains neurons to the diaphragm) can be explained by the selection of the study patients, who presented without clinically significant respiratory involvement, a prerequisite to be able to safely lay in the scanner for study purposes.

In comparison, SC gray matter reduction was larger at lower disc levels (e.g., C4/C5) projecting to the proximal arm muscles innervated by the cord segments C5/C6.

Patients with more severe disease phenotype (Type IIIa) showed more pronounced SC GM atrophy than patients with milder phenotype (Type IIIb).

Most importantly, rAMIRA-derived cervical SC GM areas consistently showed significant associations with muscle strength and with the most commonly used physiotherapeutic assessments MFM, RULM, and RHS. SC GM area at the level C3/C4 explained 32% of MFM variance and 33% of RHS variance, respectively, indicating the clinical significance of the SC GM atrophy observed in patients with SMA.

SC GM atrophy is not specific for SMA and can also be observed in other lower motor neuron diseases, as, e.g., the Post-Polio-Syndrome [25]. Comparing our results in SMA to studies conducted in patients with other motor neuron disorders involving specifically lower motor neurons (Post-Polio-Syndrome) [25] or both lower and upper motor neurons (amyotrophic lateral sclerosis (ALS)) [42], the relative reduction in GM areas compared to age- and sex-matched controls is slightly less pronounced in SMA. Differences in disease duration (PPS) and severity of pathology (ALS) might contribute to the somewhat more pronounced GM atrophy observed in these disorders.

In line with early works focusing on total cord area assessments in patients with SMA [43], we also observed significant total cord area reductions at the intervertebral disc level C3/C4, C4/C5, and C5/C6 in our study in patients compared to HCs, but these reductions were less pronounced than the SC GM atrophy observed in our study (i.e., 7% TC area reduction vs. 13% GM area reduction at the level C3/C4 in patients compared to HC). This finding is in line with the expected pathology in SMA with spinal motor neuron degeneration in GM with relative WM sparing. While El Mendili [43] et al. did not found significant associations between total cord area and measures of disability in his SMA cohort, SC GM area in our study was significantly associated with established physiotherapeutic disease metrics.

Our data confirm previously reported results by Querin et al. [26], investigating upper cervical SC GM atrophy in patients with SMA type III and IV using a T2*w sequence. rAMIRA-derived cervical SC GM area measurements thereby show smaller relative standard deviations at the corresponding levels C2/C3 to C5/C6 (7.1%−10% in HCs, 10.1%−13.7% in patients with SMA) compared to the T2*w derived metrics reported by Querin (9.5–20.8% in HCs, 17.3–25.9% in patients with SMA, derived from [26], Fig. 2). Given that the same in-plane resolution was used (0.5 × 0.5mm^2^) in both studies, this difference in precision might be explained by improved GM/WM contrast offered by the rAMIRA approach.

Our study also investigated the lower thoracic cord, a region generally prone to imaging artifacts. Patients with SMA who could be evaluated at the lumbar enlargement level using rAMIRA showed significant GM area reductions by 17% at T_max_ compared to HCs. In contrast to the cervical levels, however, no significant associations with clinical and physiotherapeutic outcomes could be detected in the thoracic segment. In view of the robust associations observed between lower thoracic cord GM area and clinical metrics in other lower motor neuron disorders [25], this finding is likely explained by two factors: first, the small sample size of our cohort and second inability to scan the most disabled patients at the thoracic level due to osteosynthesis material reducing the clinico-phenotypic variability at that level. Ongoing longitudinal studies in patients without spinal osteosynthesis material will further help to understand the dynamics and clinical relevance of lower thoracic GM atrophy in SMA. Acquisition of the axial images was perpendicular to the SC (Fig. 1C), allowing correct positioning, even in the case of severe scoliosis frequently present in patients with SMA.

There are some limitations of our study:

Due to the rarity of the disease, the study sample size is rather small, but in line with the other studies [26].

As we used the rAMIRA imaging approach recently developed at our center [23], we decided for a single-center study design to ensure the highest image quality and consistency in the acquisition of images by one experienced MRI technician. The quality of the rAMIRA images was generally high: movement artifacts only limited segmentation in three images of the HCs (2.4% of all images acquired in HCs). GM segmentation in patients with SMA was more challenging due to osteosynthesis material artifacts in 13 images (10.3%). Inclusion of more severely affected patients with SMA affects quality and reduces completeness of the data but represents the true variation of disease severity in patients with SMA.

For consistency reasons, the same rAMIRA protocol was used for the cervical and thoracic SC, although more favorable imaging conditions at the cervical SC would allow for a protocol with shorter acquisition time.

Several approaches to automatically or semi-automatically segment *cervical* SC GM areas are available and their use can potentially improve SC atrophy analyses in terms of time and operator dependency [44–47]; however, these segmentation approaches still require manual corrections. Automated segmentations methods that allow accurate segmentation of the thoracic cord gray matter are not available yet. To use a uniform segmentation algorithm in the cervical and thoracic cord, we decided to manually segment the GM, which we still consider the gold standard. This approach showed a very high inter-rater reliability.

Further methodological refinements including the development of rAMIRA-specific automatic segmentation methods also applicable to the lower thoracic cord are ongoing [25], which hopefully will facilitate larger multicentric designs in the study of SC disorders in the future.

This is a cross-sectionally designed study, and patients were partially treated with SMA modifying agents or untreated; thus, the sensitivity of the marker regarding treatment effects could not be investigated. Longitudinal studies are necessary to evaluate if GM area is sensitive to changes over time and to assess the possibility to detect therapeutic effects in drug trials.

A pilot study investigated longitudinally three adult patients with SMA under treatment with Nusinersen with T2*w MRI derived TC and GM area measurements every 4 months at the intervertebral disc level C3/C4 [48]. The TC areas were comparable with our measurements, and the GM areas were smaller, consistent with other T2*w MRI-based measurements [26]. Due to the small sample size, only trends could be reported, indicating generally stable values of SC GM and TC areas over a time period of 12 months in two treated patients and a trend towards an increased GM area in one patient [48]. The small relative standard deviation of rAMIRA-based GM metrics in both HCs and patients with SMA in our study compared to, e.g., T2*w imaging approaches [26] makes this an appealing and potentially sensitive approach to investigate even subtle alterations.

In conclusion, rAMIRA imaging is a promising approach for GM quantitation in the cervical and thoracic SC in patients with 5q-SMA in both academic and clinical settings. Our study demonstrates that SC GM atrophy is detectable in patients with 5q-SMA types II and III compared to HCs and correlates with clinical measures of disability. Further longitudinal investigations are necessary next steps to evaluate the potential of this novel and easy to assess imaging marker for monitoring the disease course and therapeutic response in patients with SMA.

## Supplementary Information

Below is the link to the electronic supplementary material.

Supplementary file1 (PDF 7 KB)

## Data Availability

Upon reasonable request, we will render the detailed results derived from the reported analyses available.
