# Supplementary figures and images for: Spinal cord gray matter atrophy is associated with disability in spinal muscular atrophy
Source: J Neurol. 2025 Jan 7;272(1):102. doi: 10.1007/s00415-024-12740-3 (PMC11706851; doi:10.1007/s00415-024-12740-3)

# Supplemental material: Flowchart

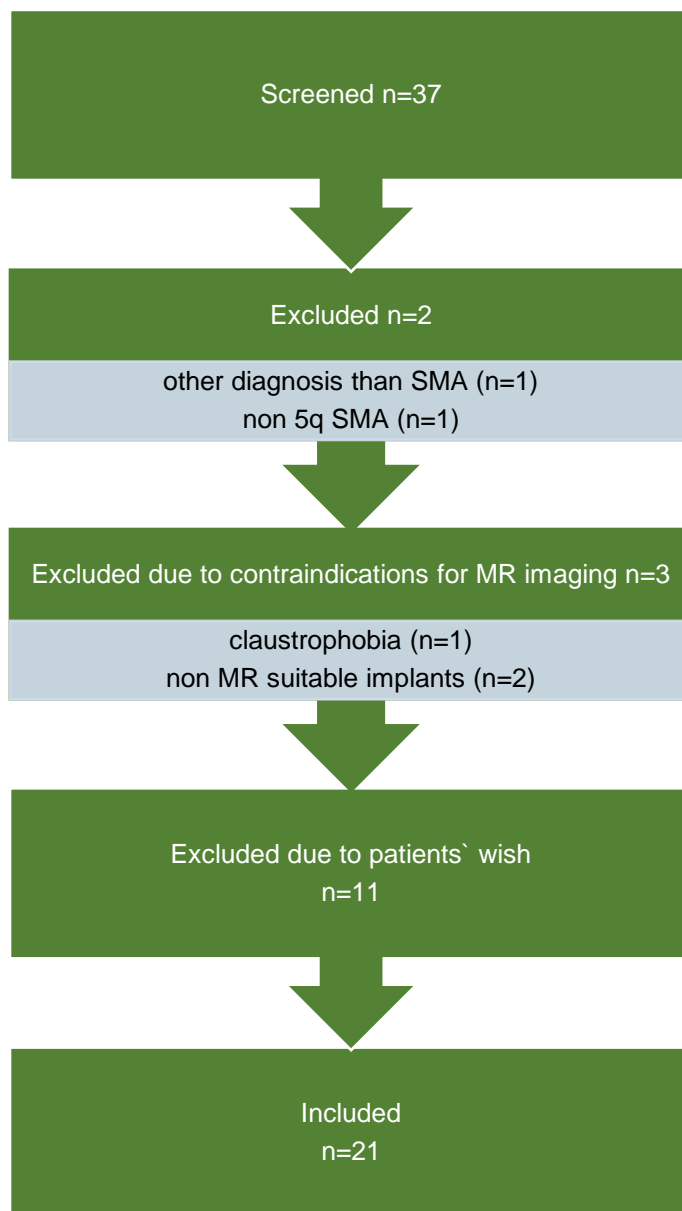

**Appendix Figure 1:** Flowchart of inclusion of SMA patients

Supplement: Supplementary file 1 — Supplementary file1 (PDF 7 KB) [file 415_2024_12740_MOESM1_ESM.pdf]
